# Endoscopic transpapillary drainage for walled-off pancreatic necrosis with complete main pancreatic duct disruption by metallic stent placement: A retrospective study

**DOI:** 10.3389/fmed.2022.1064463

**Published:** 2022-12-07

**Authors:** Yajie Meng, Jiewen Ding, Chuan Tian, Min Wang, Kejiang Tang

**Affiliations:** Department of Gastroenterology, The People’s Hospital of Nanchuan, Chongqing, China

**Keywords:** walled-off pancreatic necrosis, complete main pancreatic duct disruption, endoscopic retrograde cholangiopancreatography, endoscopic transpapillary drainage, covered self-expanding metallic stent

## Abstract

**Background:**

Walled-off pancreatic necrosis (WOPN) is a serious complication of acute necrotizing pancreatitis (ANP) and may lead to disruption of the main pancreatic duct (MPD). Endoscopic passive transpapillary drainage (PTD) is an effective method for treating MPD disruptions. However, WOPN with complete MPD disruption is usually accompanied by disconnected pancreatic duct syndrome (DPDS), especially with infected necrosis. Endoscopic PTD with a fully covered self-expanding metallic stent (FCSEMS) and a plastic stent placement may have the potential for future application in treating complete MPD disruption in patients with WOPN.

**Methods:**

Patients with WOPN caused by ANP were classified according to the 2012 Atlanta classification and definition. In all patients, ERCP was performed 2 times. First, 3 patients were diagnosed with complete MPD disruption by ERCP. At the time of diagnosis, a plastic pancreatic stent (7Fr) was placed. Second, they underwent endoscopic PTD for WOPN with complete MPD disruption in which an FCSEMS and plastic stent placement were the only access routes to the necrotic cavity.

**Results:**

The etiology of pancreatitis in these patients was of biliary, lipogenic, and alcoholic origin. The WOPN lesion size ranged from 6.5 to 10.2 cm in this study, and the type of WOPN was mixed in two cases and central in one case. The type of MPD disruption was complete in all three patients. The locations of disruption included the pancreatic body and head. The time from occurrence to the first ERCP was 18, 23, and 26 days, respectively. The main symptoms were abdominal pain, abdominal distention, fever, gastrointestinal obstruction, and/or weight loss. The three patients with symptomatic WOPN and MPD disruption underwent endoscopic PTD with FCSEMS and plastic pancreatic stent placement. Technical and therapeutic successes were achieved in 3/3 of patients. The mean time of stenting was 28–93 days. The clinical symptoms connected with WOPN and collection disappeared postoperatively in all three patients. During the follow-up period of 4–18 months, no patient developed collection recurrence or other complications, such as gastrointestinal bleeding or reinfection. All patients recovered uneventfully.

**Conclusion:**

In patients with WOPN with complete MPD disruption, endoscopic PTD with FCSEMSs and plastic stent placement may be an effective and safe method of treatment.

## Introduction

Walled-off pancreatic necrosis (WOPN) is a serious complication of acute necrotizing pancreatitis (ANP) and may lead to disruption of the main pancreatic duct (MPD) (1, 2). MPD disruption occurs in 38% of patients with WOPN and includes partial and complete disruption, with complete MPD disruption being documented in 43.8% of cases (2).

Endoscopic treatment of pancreatic duct disruption mainly includes passive transmural drainage, passive transpapillary drainage (PTD), or a combination of both techniques (3). Endoscopic PTD consists of endoscopic sphincterotomy and stenting of the MPD to ensure the physiological outflow of pancreatic juices into the duodenum. PTD is an effective method of treating MPD disruptions. However, WOPN with complete MPD disruption is usually accompanied by disconnected pancreatic duct syndrome (DPDS), especially with infected necrosis, and the selection of optimal treatment remains challenging (2–4). Sometimes, additional percutaneous drainage for persistent necrotic collections is required (5).

In this study, we aimed to retrospectively review our own experience with patients with WOPN and complete MPD disruption in the head or neck of the pancreas and who underwent endoscopic PTD with a fully covered self-expanding metallic stent (FCSEMS) and a plastic stent as the only access route to the necrotic cavity to analyze the efficacy and safety of this method. To the best of our knowledge, this method has not been reported in the literature and may have the potential for future application in treating complete MPD disruption in patients with WOPN.

## Materials and methods

The study was approved by the Ethics Committee of our hospital. All patients gave their informed consent for the endoscopic procedures. In particular, the FCSEMS was placed in the three patients after a multidisciplinary and ethical review and the patient’s personal choice.

### Patients

We retrospectively investigated three patients with WOPN and complete disruption MPD who underwent PTD with FCSEMS and plastic stent placement from January 2018 to January 2021 in the People’s Hospital of Nanchuan, Chongqing, China.

Walled-off pancreatic necrosis and the complete MPD disruption were diagnosed by contrast-enhanced computed tomography (CECT), magnetic resonance cholangiopancreatography (MRCP), endoscopic ultrasound (EUS), and endoscopic retrograde cholangiopancreatography (ERCP) in our center (6). WOPN caused by ANP was classified according to the 2012 Atlanta classification and definition (7).

**FIGURE 1:**
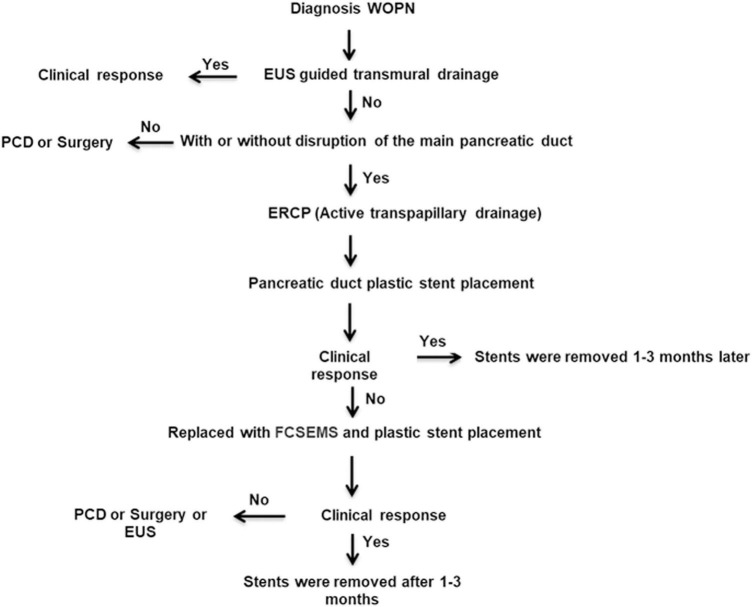
Algorithmic approach to the management of walled-off pancreatic necrosis with complete main pancreatic duct disruption by endoscopic transpapillary drainage with fully covered self-expanding metallic stent and plastic stent placement in our center. WOPN, walled-off pancreatic necrosis; EUS, endoscopic ultrasound; PCD, percutaneous catheter drainage; ERCP, endoscopic retrograde cholangiopancreatography; FCSEMS, fully covered self-expanding metallic stent.

The indications for PTD with FCSEMS and plastic stent placement were as follows: (1) inability to undergo EUS-guided endoscopic transmural drainage from the gastrointestinal tract wall, with no communication with the MPD revealed by endoscopic retrograde pancreatography (ERP); (2) WOPN lesion diameter greater than 5 cm, accompanied by one or more symptoms, such as abdominal pain or obstruction of the gastric outlet, intestinal system, or biliary system; (3) suspected infection of WOPN based on the patient’s clinical course (fever and leukocytosis) despite the administration of intravenous antibiotics and the presence of gas within the collection as observed on CECT; (4) complete MPD disruption and a rupture site located in the head or body of the pancreas; and (5) ineffective drainage by the first ERCP for plastic stenting of the pancreatic duct with no relief of fever symptoms, followed by replacement with an FCSEMS and plastic pancreatic duct stent. The flow of the study is presented in Figure 1.

### Procedures

All ERCP-related procedures and EUS-related assessments were performed by the same endoscopist (who performs more than 200 ERCP procedures every year). Linear EUS was performed with a linear EUS device (SU7000, EG-530UT, Fujifilm, Tokyo, Japan) at a frequency of 7.5 MHz. ERCP was performed with a duodenoscope (ED-250XL5, Fujifilm, Tokyo, Japan) under general anesthesia. All patients received antibiotics before the procedure (piperacillin combined with moxifloxacin). Routinely, antibiotic treatment was continued for 1 week or until the patient’s temperature was normal for more than 5 days.

**FIGURE 2:**
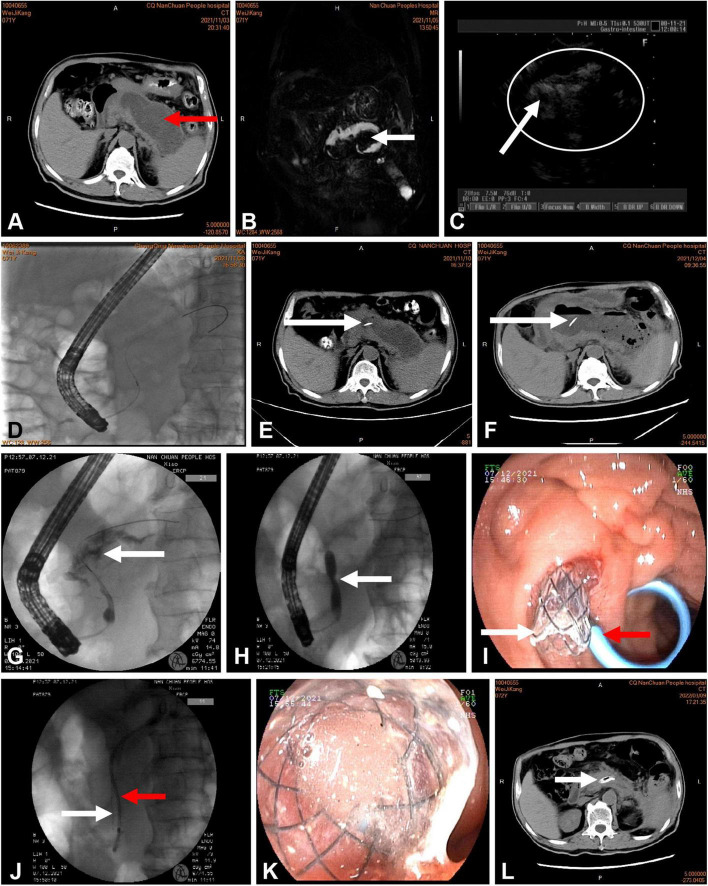
**(A)** Computed tomography (CT) of the abdomen showing more than 50% pancreatic parenchymal necrosis and pancreatic fluid collection 102 mm in diameter (red arrow). **(B,C)** Rich debris was present in the cavity, as shown by magnetic resonance cholangiopancreatography (MRCP) (white arrow) and endoscopic ultrasound (white circle). **(D)** Endoscopic retrograde cholangiopancreatography (ERCP) showing a disrupted pancreatic duct communicating with the WOPN. **(E,F)** CT showing a plastic stent (white arrow) communicating with the WOPN. **(F)** The cavity was not reduced at 4 weeks after intervention. **(G)** ERCP showing extravasation of contrast material (white arrow) from the region of the pancreatic neck. **(H)** Balloon dilation of the pancreatic duct stenosis (white arrow). **(I,J)** Deployment of a metal stent (white arrow) and a plastic pancreatic stent (red arrow) inside the WOPN. **(K)** Drainage of necrotic tissue and fluid from the metallic stent. **(L)** CT performed 3 months after the end of active drainage. In the main pancreatic duct, the transpapillary metal and plastic pancreatic stents were visible.

The technique for PTD involved the following steps (Figure 2). In patients who underwent active transpapillary drainage, sphincterotomy was performed during ERCP, and an aspirate from the collection was sent for amylase activity analysis and microbial culture; subsequently, the antibiotic therapy was modified as appropriate according to the culture results. This was followed by mechanical dilatation of the MPD using a bougie-type 7-Fr dilator (Wilson-Cook Medical, Bloomington, IN, USA). Pancreatic endoprostheses (7-Fr Pancreatic Stent, Wilson-Cook Medical, Bloomington, IN, USA) were introduced through the papilla. The distal end of the pancreatic endoprosthesis was located at the site of MPD disruption. The WOPN lesion size was monitored every week by transabdominal ultrasonography or abdominal CT. After 1 week of postoperative intravenous antibiotic treatment, the patients were still feverish; 4 weeks later, the cyst was enlarged in one patient and the same size in the other two patients. The reason for these results was probably the blockage of the thin 7-Fr stents. However, for WOPN, there was still a chance of blockage reoccurrence after replacing the 7-Fr plastic stents. Drainage by minimally invasive surgery or percutaneous catheters is an important method for resolving WOPN. However, these three patients had been strictly screened. After a multidisciplinary and ethical review and by the patients’ personal choice, the 7-Fr plastic stent was placed with an FCSEMS (8 mm × 8 cm, Micro-Tech, Nanjing, China). However, before replacing the original plastic stent with the FCSEMS, balloon dilation was performed because all three patients had a pancreatic ductal stricture distal to the disruption. An expansion balloon with a diameter of 4–6 mm was first placed along the guide wire to expand the sphincter of the pancreatic duct and the narrowed pancreatic duct. Then, the pancreatic duct double guide-wire method was used. First, the FCSEMS was placed along the guide wire and then a 5-Fr plastic pancreatic duct stent was placed along the other guide wire.

#### Outcome definitions

The therapeutic success of WOPN endotherapy was defined as the absence of symptoms and complete regression of the collection or a collection size < 40 mm on CT, with no need for surgery. Recurrence of WOPN was defined as collection size > 40 mm on imaging examinations or recurrence of symptoms during follow-up (3, 8). Any complications occurring after PTD and the corresponding outcomes were also retrieved.

## Results

### Patient characteristics

The mean age of the three patients was 66.3 years; two patients were men and one patient was a woman. The etiology of pancreatitis in these three patients was of biliary, lipogenic, and alcoholic origin. The WOPN lesion size ranged from 6.5 to 10.2 cm. The type of WOPN was mixed in two cases and central in one case. The MPD disruption type was complete in all three patients. The locations of disruption include the pancreatic body and head. The time from occurrence to the first ERCP was 18, 23, and 26 days, respectively. The main symptoms were abdominal pain, abdominal distention, fever, gastrointestinal obstruction, and or weight loss. The characteristics of the three patients are presented in Table 1.

**TABLE 1:** Characteristics of the three patients with walled-off pancreatic necrosis (WOPN) and complete disruption of the main pancreatic duct.

Characteristics	Case 1	Case 2	Case 3
Sex	Male	Female	Male
Age (year)	69	58	72
Etiology	Alcoholic	Biliary origin	Lipogenic
WOPN size (cm)	8.3	6.5	10.2
WOPN type	Mixed necrosis (pancreatic and peripancreatic)	Central necrosis (pancreatic)	Mixed necrosis (pancreatic and peripancreatic)
MPD disruption type	Complete	Complete	Complete
MPD disruption location	Body	Head	Head
Time from occurrence of acute pancreatitis (days)	18	23	26
Main symptoms indicating endotherapy	Abdominal pain; abdominal distention; fever; gastrointestinal obstruction	Abdominal pain; abdominal distention; gastrointestinal obstruction	Fever; abdominal distention; gastrointestinal obstruction; weight loss

### Technical success

Technical and therapeutic successes were achieved in all 3 patients. ERCP was performed 2 times in all 3 patients. First, the 3 patients were diagnosed with complete MPD disruption, and ERCP revealed contrast leakage to the necrotic collection in the region of the pancreatic head and neck. At the time of diagnosis, a plastic pancreatic stent (7-Fr) was placed. In the three patients, the clinical symptoms of abdominal distention, fever, and gastrointestinal obstruction, which are associated with WOPN and collection, disappeared during follow-up. Among the three cases, 2 patients were suspected to have complications due to infections before the operation because of positive microbial culture results. One patient was treated with an FCSEMS combined with a plastic stent 1 month after the first ERCP procedure in which a plastic stent was placed in the pancreatic duct for drainage due to treatment failure. All three patients with symptomatic WOPN and complete MPD disruption underwent endoscopic PTD with FCSEMS and plastic pancreatic stent placement.

### Complications and follow-up

There were no complications in any of the three patients. The pancreatic duct stents of the three patients were actively removed after a mean stenting duration of 28–93 days. During the follow-up period of 4–18 months, no patient developed collection recurrence or other complications, such as gastrointestinal bleeding or reinfection. The recovery was uneventful for all patients. We will continue to closely monitor the clinical symptoms and recovery of the patients.

In some patients with WOPN with complete MPD disruption, endoscopic PTD with FCSEMSs and plastic stent placement may be an effective and safe method of treatment.

## Discussion

In patients with WOPN with complete MPD disruption who cannot undergo transmural drainage, transpapillary access may be an effective and safe method of treatment (9). However, approximately 20% of patients still exhibit poor results and experience recurrence (3). The stents used for WOPN with PTD are all plastic stents, and there may be poor drainage because the diameter of the stents is 5–10 Fr (10, 11). Our three patients were diagnosed with complete MPD disruption after the initial ERCP procedure and a 7-Fr plastic stent was placed but considered ineffective. It has been reported in the literature that FCSEMS placement can be used to treat pancreatic duct stenosis (12). For our three patients, we tried to replace the plastic stent with an FCSEMS with a diameter of 8 mm. Intraoperatively, a large amount of necrotic tissue and fluid flowed out from the stent. Postoperative follow-up revealed effective resolution of the necrotic tissue exudation.

Considering that the FCSEMS was placed in the pancreatic duct, it was confirmed that there was no serious expansion of the pancreatic duct in the head or neck of the pancreas. Additionally, the placement of an FCSEMS may block the pancreatic duct branches and increase the risk of pancreatitis. Therefore, we intraoperatively placed a plastic pancreatic duct stent concomitant with FCSEMS, and the pancreatic fluid could be drained through the plastic pancreatic duct stent, reducing the occurrence of postoperative pancreatitis. Our experience confirms this hypothesis, as there were no cases of post-ERCP pancreatitis.

Based on the treatment course of our three patients, we hypothesized that for WOPN with complete MPD disruption not located in the tail of the pancreas in which drainage cannot be performed through the gastric or duodenal wall, transpapillary FCSEMS placement would be an alternative option when PTD with a plastic stent is ineffective or complicated by infection.

Despite these findings, there is still much uncertainty about the optimal therapeutic approach, and there are some limitations to this study. Due to the small number of cases, it is unknown whether the metallic stent placed can be substituted for a plastic stent when transpapillary plastic stent drainage is not effective. The optimal timing for metal stent extraction remains unclear. Moreover, it is unknown if it is necessary to place a plastic stent in parallel with a fully-covered metallic stent. In addition, the safety aspects of the procedure (although no complications, such as bleeding, pancreatitis, or infection, occurred in our three patients) require further exploration.

## Conclusion

In some patients with WOPN with complete MPD disruption, endoscopic PTD by FCSEMS and plastic stent placement may be an effective and safe method of treatment. However, there are still many limitations, and more summaries of practical clinical applications are needed in the future to verify the safety and efficacy of this method.

## Data availability statement

The original contributions presented in this study are included in this article/supplementary material, further inquiries can be directed to the corresponding authors.

## Ethics statement

The studies involving human participants were reviewed and approved by the Ethics Committee of the People’s Hospital of Nanchuan. The patients/participants provided their written informed consent to participate in this study. Written informed consent was obtained from the individual(s) for the publication of any potentially identifiable images or data included in this article.

## Author contributions

YM and MW designed and performed the study. JD and KT wrote the manuscript, performed the research, and analyzed the data. CT made critical revisions related to the important intellectual content of the manuscript. All authors read and approved the final version to be published.
